# First use of augmented reality headset in minimally invasive general surgery: seeing is believing

**DOI:** 10.1007/s00464-025-11985-x

**Published:** 2025-07-10

**Authors:** Ryan C. Broderick, Graham J. Spurzem, J. Jeffery Reeves, Hannah M. Hollandsworth, Bryan J. Sandler, Garth R. Jacobsen, Christopher A. Longhurst, Santiago Horgan

**Affiliations:** 1 Division of Minimally Invasive Surgery, Department of Surgery, Center for the Future of Surgery, University of California San Diego, 9300 Campus Point Dr. La Jolla, San Diego, CA 92037 USA; 2 Joan & Irwin Jacobs Center for Health Innovation, University of California San Diego, San Diego, CA USA

**Keywords:** Augmented reality, Spatial computing, Minimally invasive surgery, Laparoscopy, Endoscopy

## Abstract

**Background:**

Augmented reality (AR) is an evolving technology with the potential to transform surgical practice. By superimposing digital information onto the surgeon’s field of view, AR headsets provide an unobstructed view of the minimally invasive operative field, eliminating the need to divert attention to external monitors. We present the first series of minimally invasive general surgery (MIS) cases performed using the Apple Vision Pro headset in the USA.

**Methods:**

Attending surgeons and trainees performed consecutive MIS cases at our institution while wearing the headset from August 2024 to December 2024. Using open-source software, laparoscopic/endoscopic video sources were displayed as virtual monitors in the physical operating room space. The virtual monitors served as the primary monitors through which surgeons performed each case. Standard monitors remained present to enable other members of the operative team to view the operations. At the conclusion of each case, the operating surgeon completed the NASA Task Load Index (NASA-TLX) assessment tool to evaluate perceived workload while operating with the headset. 30-day perioperative complications were also assessed.

**Results:**

A total of 41 MIS cases were performed by 3 attending surgeons and 4 trainees. The most common procedure was laparoscopic sleeve gastrectomy (N = 9, 22.0%). Open-source software enabled simultaneous viewing of up to 3 virtual displays that could be individually positioned in the surgeon’s visual field. The mean NASA-TLX score for all participants was 22.3 ± 4.7, indicating a low perceived intraoperative workload. There was no significant difference in NASA-TLX scores between attending surgeons and trainees (19.8 ± 5.3 vs 24.8 ± 3.0, p = .23). There were no intraoperative complications; 30-day morbidity and mortality were 0%.

**Conclusion:**

This study serves as a proof of concept for the use of an augmented reality headset in minimally invasive general surgery.

Minimally invasive surgery (MIS) is widely accepted as the standard of care for numerous procedures. Significant technological advances have been made since the inception of MIS, from novel laparoscopic instruments to robotic surgical platforms and advanced camera technology. [1] More recently, the integration of extended reality (XR) platforms and spatial computing has enabled advances in minimally invasive surgical practice, particularly in spine surgery and neurosurgery. [2, 3] The term XR refers to the combination of real and virtual elements using computer technology, including augmented reality (AR), virtual reality (VR), and mixed reality (MR). The concept of spatial computing encompasses these terms and refers to the combined software and hardware capabilities that allow digital devices to interact with the physical world. These platforms have several other potential applications in surgical education, training, and ergonomics. [4–7]

In February 2024, Apple released its headset termed the Apple Vision Pro (AVP). AVP is a VR device with an added video see-through capability, permitting AVP to simultaneously function as an AR headset. The AR feature is enabled by streaming a view of the outside world via external-facing cameras into the screens within the headset. This capability allows the user to augment their view of the real world with various virtual features, such as preoperative imaging and 3D renderings of unique patient anatomy. To date, clinical uses of AVP during surgery in the literature are limited to procedure planning and imaging review. There have been no published uses of the headset to complete laparoscopic or endoscopic procedures in the USA. [8]

The aim of this study was to evaluate the feasibility of using a virtual monitor overlaid in physical space to perform minimally invasive general surgery procedures. Our first objective was to transmit laparoscopic and endoscopic video sources from their respective control units into AVP as virtual displays, enabling the user to perform surgery with the virtual display acting as the primary surgical monitor. The second objective was to evaluate the perceived mental workload of surgeons operating with the headset and assess short-term perioperative patient outcomes.

## Methods

### Operating room configuration

**Fig. 1:**
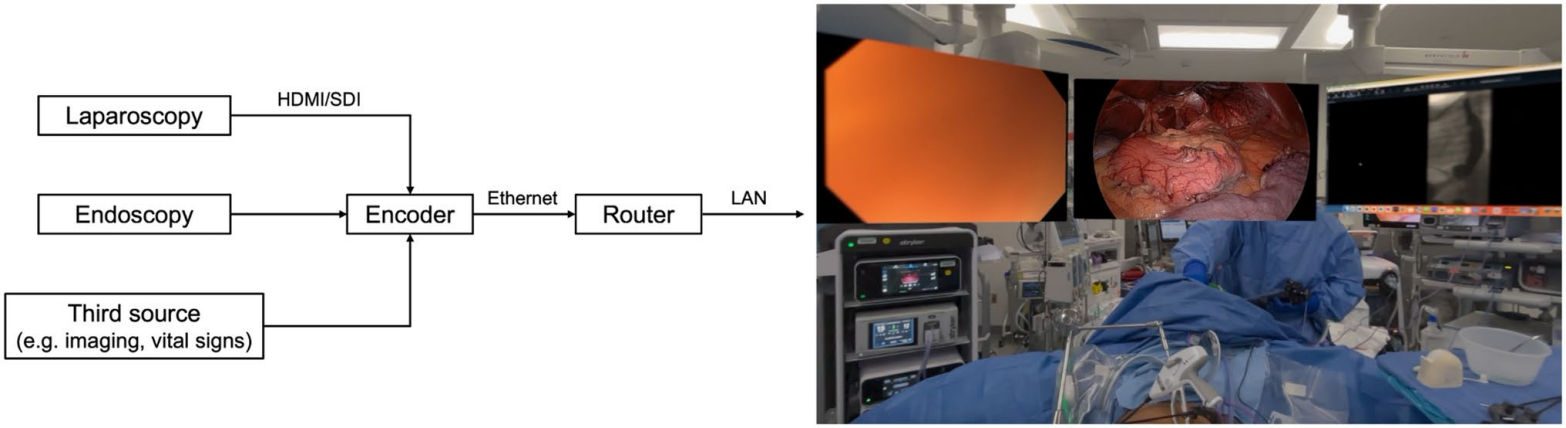
Operating room configuration used to transmit video sources to open-source application software on Apple Vision Pro. Image shows the endoscopic (left), laparoscopic (center), and preoperative imaging (right) video sources as virtual displays overlaid in the physical operating room environment

The configuration used to transmit operating room (OR) video sources to application software on the headset is shown in Fig. 1. Video sources from standard laparoscopic (Stryker 1788 platform, Kalamazoo, MI) and endoscopic (Olympus EVIS X1 endoscopy system, Center Valley, PA) control units were diverted to commercially available video encoders via HDMI/SDI as needed. Our configuration enables transmission of an optional third video source based on surgeon preference (e.g., preoperative imaging or vital signs from the anesthesia monitor). From the encoders, video sources were then transmitted via ethernet to a high-bandwidth router capable of propagating 4K video streams via a local area network (LAN). After connecting the headset to the LAN, video sources were then viewed using an open-source application capable of detecting the encoded sources and displaying them as individual virtual displays. These virtual displays showing the laparoscopic and endoscopic video sources were then viewed in the headset and used to perform surgery. Standard surgical monitors remained present in the OR for surgical assistants, technologists, and anesthesia staff to view the operations.

### Study design

An institutional review board-approved prospective study was conducted from August 2024 to December 2024 at our academic center. Informed consent was obtained from patients undergoing surgery and surgeons operating with the headset to participate in the study. Consecutive MIS cases were performed by attending surgeons, minimally invasive surgery fellows, and general surgery residents while wearing the headset. Patient demographics including age, gender, and body mass index (BMI) were collected. The number and types of procedures performed using the headset were also tabulated.

### Outcomes

At the conclusion of each procedure, the operating surgeon completed the National Aeronautics and Space Administration Task Load Index (NASA-TLX) assessment on a mobile application. NASA-TLX is a subjective assessment tool that evaluates operators working with human–machine interface systems and was developed to measure domains of subjective workload in high-risk industries, including healthcare. [9, 10] The assessment provides an overall workload score (scaled to 0 = low, 100 = high) based on a weighted average of ratings on six subscales: mental demand, physical demand, temporal demand, performance, effort, and frustration. [11, 12] Perioperative patient outcomes including the number of intraoperative complications and 30-day morbidity and mortality were also assessed.

### Statistical analysis

Categorical variables were reported as a frequency and percentage. Continuous variables were reported as a mean and standard deviation (SD). Mean NASA-TLX scores between attending surgeons and trainees were compared using an independent sample *t* test. Analysis was performed using R (Version 4.4.1, Vienna, Austria).

## Results

### Operating room workflow

Surgeons donned the AR headset prior to scrubbing and performed eye tracking and hand calibration using the headset’s internal calibration functionality. Surgeons then performed the entirety of each operation using the virtual display in the headset as the primary surgical monitor. Our video encoder configuration enabled simultaneous viewing of up to three video sources, each appearing as its own virtual display. The open-source video application software allowed each display to be individually repositioned and resized in the surgeon’s virtual visual field. Once positioned, the displays remained fixed in space until moved by the surgeon. This allowed the virtual displays to be positioned in any location within the OR based on surgeon preference. The headset also provided an unobstructed view of the external operative field, allowing the surgeon to place trocars, change laparoscopes, and close skin at the conclusion of the case. Use of the headset also eliminated the need for external surgical monitors to be present at the head of the OR table during foregut and bariatric surgery procedures. Multiple headsets could connect to the same LAN, providing simultaneous viewing of the same video sources by multiple surgeons, each through their own uniquely positioned virtual monitors.

### Patient demographics and operative data

A total of 41 minimally invasive procedures were performed with the headset acting as the primary surgical monitor. There was no perceived latency or lag of video feeds during surgery. Average patient age was 56.1 ± 15.6 years, 56.4% of patients were female, and mean BMI was 28.8 kg/m^2^. A variety of foregut, bariatric, endoscopic, and general surgery procedures were performed (Table 1). The most common procedure was laparoscopic sleeve gastrectomy (N = 9, 22.0%), followed by laparoscopic cholecystectomy with indocyanine green cholangiography (N = 8, 19.5%), and laparoscopic hiatal hernia repair with Nissen fundoplication (N = 7, 17.1%). The longest procedure performed was a laparoscopic subtotal gastrectomy with D2 lymphadenectomy and intraoperative upper endoscopy, lasting 121 min. Procedures lasting around this timeframe required a battery change or plug-in to a charger.

**Table 1:** Procedures performed with virtual displays on Apple Vision Pro acting as the primary surgical monitor

Procedure, N (%)	Total (N = 41)	Operative time (min), mean (SD)^*^
Laparoscopic sleeve gastrectomy, intraoperative upper endoscopy	9 (22.0)	61.3 (15.5) [59.2 – 70.5] [13]
Laparoscopic cholecystectomy with indocyanine green cholangiography	8 (19.5)	49.7 (3.6) [13.0 – 289.0] [14]
Laparoscopic hiatal hernia repair with Nissen fundoplication, intraoperative upper endoscopy	7 (17.1)	60.0 (10.6) [86.0 – 160.0] [15]
Laparoscopic inguinal hernia repair with mesh	5 (12.2)	38.8 (3.3) [21.0 – 201.0] [16]
Laparoscopic hiatal hernia repair, Linx procedure, intraoperative upper endoscopy	3 (7.3)	52.0 (15.4) [31.0 – 159.0] [17]
Upper endoscopy with balloon dilation	3 (7.3)	11.3 (1.5)
Laparoscopic adrenalectomy	2 (4.9)	48.5 (4.9) [83.0 – 240.0] [18]
Laparoscopic gastrojejunostomy, intraoperative upper endoscopy	1 (2.4)	61.0
Laparoscopic subtotal gastrectomy, D2 lymphadenectomy, intraoperative upper endoscopy	1 (2.4)	121.0
Laparoscopic hiatal hernia repair, endoscopic gastric fundoplication	1 (2.4)	63.0
Laparoscopic partial sleeve gastrectomy with resection of gastric gastrointestinal stromal tumor, intraoperative upper endoscopy	1 (2.4)	90.0

^*^ Reference ranges for operative time in standard laparoscopic cases noted in brackets

### Outcomes

A total of 3 attending surgeons, 1 minimally invasive surgery fellow, and 3 general surgery residents performed surgery while wearing the headset. The mean NASA-TLX score for all participants was 22.3 ± 4.7, indicating a low perceived intraoperative workload. There was no significant difference in mean NASA-TLX scores between attending surgeons and trainees (19.8 ± 5.3 vs 24.8 ± 3.0, p = 0.23). There were no intraoperative complications or device malfunctions; 30-day morbidity and mortality were both 0%.

## Discussion

In this study, we report a series of laparoscopic and endoscopic procedures performed with Apple Vision Pro. We demonstrate that operating with the headset is feasible, with a low perceived mental workload experienced by surgeons across a range of training levels. The headset effectively acted as a standalone surgical monitor, enabling surgeons to view multiple 4K video sources simultaneously with no perceived latency. The attending surgeons and surgical residents who participated in this study reported several subjective benefits of the headset not captured by NASA-TLX or other measures. The ability to place the virtual displays in any location within the OR permitted surgeons to operate in the position of their choice without ergonomic constraints. Viewing patient vital signs in real time alongside the operative video sources also enabled surgeons to quickly and effectively communicate with the anesthesia team regarding blood pressure changes requiring intervention. In addition, readily correlating preoperative imaging with findings in the operative field was useful for navigating complex anatomy. Surgeons found the headset to be particularly advantageous during procedures requiring intraoperative upper endoscopy, as the laparoscopic and endoscopic video sources could be easily viewed side by side without needing to physically maneuver OR equipment. This enabled surgeons to more comfortably utilize intraoperative endoscopy for coordination during critical portions of procedures, such as creating an esophagojejunostomy, especially in ORs with limited space. Overall, the benefits afforded by the headset positively influenced the operative experience for surgeons.

AR platforms have demonstrated utility in various surgical disciplines, predominantly in those involving rigid structures. For example, numerous head-mounted display devices have been utilized in spine surgery to assist with pedicle screw placement and other percutaneous interventions. [19] AR technology is also influencing the landscape of orthopedic hip surgery, from total hip arthroplasty to the management of hip fractures. [20] In contrast, there is limited literature on the use of AR technology in laparoscopic surgery, as the relative non-rigidity of abdominal structures complicates any augmentation of the laparoscopic field. [21] Laparoscopic liver surgery, particularly oncologic resection, represents a promising area for AR applications. [22, 23] AVP may have the potential to further progress AR integration in laparoscopic surgery and is gaining attention worldwide. In May 2024, surgeons in Chennai, India reported completing at least 30 laparoscopic procedures using AVP in a similar manner as this study. [24]

While our use of the headset in minimally invasive surgery remains limited to video source transmission, there is substantial opportunity for application software development and engineering solutions to harness the potential of this technology in surgery. Intraoperative teaching requires effective and precise communication between faculty and trainees. However, traditional instruction of trainees during MIS is primarily performed verbally, as pointing at structures on the operative screen is often ineffective and risks compromising sterility. Telestration systems capable of enabling real-time freehand annotation over operative video are available for laparoscopic and robotic-assisted surgery and are gaining popularity for surgical education. [25] Development of virtual telestration applications compatible with AR headsets could enhance current intraoperative teaching practices and open additional avenues for surgical telementoring and proctoring. [26] The role of AR in immersive surgical simulation training is also expanding and could serve as an effective platform for trainees to hone their skills in a low-risk environment. [27] Expert surgeons may also benefit from this type of simulation technology for preoperative planning and rehearsal. In addition, the integration of 3D cameras and renderings of patient imaging with AR technology could provide intraoperative guidance to augment decision-making. Collaborative efforts between surgical educators and engineers will be critical in the development of these programs.

The use of AR headsets also has ergonomic implications for surgeons. It is widely recognized that appropriate positioning of the surgical monitor is essential to achieve optimal ergonomics during laparoscopic surgery. [28] MIS surgeons often hold their neck and shoulders in static positions for prolonged periods and are significantly more likely to experience musculoskeletal pain than surgeons performing open procedures. These work-related injuries can have detrimental effects on surgeon well-being, productivity, and longevity. [29] To keep the neck in an ergonomically favorable position, monitors should be placed directly in front of the surgeon at a height such that the surgeon’s eye level and the center of the monitor are less than 30 degrees. [30] While there may be patient factors or physical limitations of the operating room itself that prohibit optimal ergonomics, the surgeon’s ability to readily change the position of their monitor with the headset to any location circumvents this issue. However, the weight of AVP at roughly 600 g is significant and may place additional stress on the head and cervical spine if not positioned appropriately. [31] It remains to be seen if future generations of AR headsets in the marketplace will provide a lighter alternative with equivalent functionality. 

AR technology also has the potential to transform operating room layouts, workflows, and equipment requirements. While AVP carries a $3500 price tag, a single headset is cheaper than a fleet of standard surgical monitors and has a much smaller physical footprint. Standard monitors in the operating room will always be required for those without a headset to view an operation, but it is possible that substituting a fraction of standard monitors for headsets may reduce clutter and provide a net cost benefit. This substitution could be particularly useful in low-resource areas with limited operating space. Decluttering operating rooms more generally can also help to streamline intraoperative workflows and improve patient care. [32, 33] Future operating rooms may leverage AR technology to create a centralized display that combines real-time patient data and operative video sources, eliminating the need for several monitors to display different aspects of patient information. [34] It is feasible that consolidating patient information into a single source may streamline intraoperative workflows and improve communication between members of the operative team.

This study is limited by its single-center design and small sample size. Generalizability may also be limited by the cost of the headset and the hardware needed to transmit OR video sources to the open-source video software.

## Conclusion

This study serves as a proof of concept for the use of an augmented reality headset in minimally invasive general surgery.
